# Effects of the *pyrE* deletion mutant from *Bacillus thuringiensis* on gut microbiota and immune response of *Spodoptera exigua*

**DOI:** 10.3389/fmicb.2023.1182699

**Published:** 2023-06-02

**Authors:** Dan Zhao, Han Wu, Yazi Li, Qian Wang, Yujie Ji, Xiaochang Guo, Wei Guo

**Affiliations:** ^1^College of Plant Protection, Hebei Agricultural University, Baoding, China; ^2^Graduate School of Chinese Academy of Agricultural Sciences, Beijing, China

**Keywords:** Bt GS57△*pyrE*, uracil, gut microbiota, immune gene, *Spodoptera exigua*

## Abstract

The gut microbiota is essential for the growth and development of insects, and the intestinal immune system plays a critical role in regulating the homeostasis of intestinal microorganisms and their interactions with pathogenic bacteria. Infection with *Bacillus thuringiensis* (Bt) can disrupt the gut microbiota of insects, but the regulatory factors governing the interaction between Bt and gut bacteria are not well understood. Uracil secreted by exogenous pathogenic bacteria can activate DUOX-mediated reactive oxygen species (ROS) production, which helps maintain intestinal microbial homeostasis and immune balance. To elucidate the regulatory genes involved in the interaction between Bt and gut microbiota, we investigate the effects of uracil derived from Bt on gut microbiota, and host immunity using a uracil deficient Bt strain (Bt GS57△*pyrE*) obtained by homologous recombination. We analyze the biological characteristics of the uracil deficient strain and found that the deletion of uracil in Bt GS57 strain changed the diversity of gut bacteria in *Spodoptera exigua*, as investigated using Illumina HiSeq sequencing. Furthermore, qRT-PCR analysis showed that compared with Bt GS57 (control), the expression of the SeDuox gene and the level of ROS were significantly decreased after feeding with Bt GS57△*pyrE*. Adding uracil to Bt GS57△*pyrE* restored the expression level of DUOX and ROS to a higher level. Additionally, we observed that *PGRP-SA*, *attacin, defensin* and *ceropin* genes were significant different in the midgut of *S. exigua* infected by Bt GS57 and Bt GS57△*pyrE*, with a trend of increasing first and then decreasing. These results suggest that uracil regulates and activates the DUOX-ROS system, affects the expression of antimicrobial peptide genes, and disturb intestinal microbial homeostasis. We preliminarily speculate that uracil is a key factor in the interaction between Bt and gut microbiota, and these findings provide a theoretical basis for clarifying the interaction between Bt, host, and intestinal microorganisms, as well as for gaining new insights into the insecticidal mechanism of *B. thuringiensis* in insects.

## Introduction

Insects harbor complex microbial communities in their gut, which form a diverse microbial ecosystem that plays a critical role in the host’s physiology and resistance against pathogens (Shin et al., 2011; Kamareddine et al., 2018; Bai et al., 2021). The insect immune response is responsible for maintaining gut microbial homeostasis and plays a crucial role in regulating the interactions between commensal and pathogenic bacteria (Wu et al., 2016; Gao et al., 2020). Reactive oxygen species (ROS) and antimicrobial peptides (AMPs) are important components of the insect intestinal immunity that regulate pathogen growth and protect commensal bacteria (Bae et al., 2010; Ryu et al., 2010). ROS are primarily produced by dual oxidases (DUOX), while the immune-deficiency pathways generate AMPs (Ha et al., 2005, 2009; Zeng et al., 2022).

Recent studies have revealed that intestinal microorganisms can impact the insecticidal activity of pathogenic bacteriaused to control pests. For instance, elimination of indigenous midgut bacteria by oral administration of antibiotics has been shown to reduce the efficacy of *Bacillus thuringiensis* (Bt) against *Lymantria dispar* larvae (Broderick et al., 2006). Additionally, in some insects, the entry of Bt into the insect leads to the transformation of beneficial *bacteria* into pathogenic bacteria, thereby affecting the sensitivity of insects to Bt (Broderick et al., 2009). However, Raymond et al. demonstrated that antibiotics have an effect on the activity of Bt, and a series of experiments have shown that midgut bacteria are not essential for Bt to exert its insecticidal activity (Raymond et al., 2009). Entomopathogenic fungi have also been shown to downregulate *DUOX* expression in the midgut, leading to an imbalance in the intestinal flora that can cause the opportunistic pathogen *Serratia marcescens* to proliferate and transfer to the insect’s hemocoel, ultimately promoting mosquito death (Wei et al., 2017).

Free nucleobases are not normally present intraor extracellularly in bacteria, However, uracil was observed in *Escherichia coli* and the release of uracil is likely influenced by various environmental conditions (Rinas et al., 1995). In *Pseudomonas aeruginosa*, uracil auxotrophic mutant strains decreased virulence and biofilm formation (Ueda et al., 2009), such uracil-modulated bacterial responses were speculated that potentially threatening to host fitness (Attila et al., 2009). It has been shown that pathogenic bacteria can release uracil, which activate DUOX-dependent gut immunity, that plays an essential role in host protection against pathogen infection (Lee et al., 2013, 2015). Uracil released by allochthonous bacteria, but not by symbiotic resident bacteria, helps the host to distinguish intestinal symbiotic bacteria from pathogenic bacteria, thereby maintaining intestinal microbial homeostasis and immune balance (Lee et al., 2013). The *car* and *pyr* gene (such as *carA*, *carB*, *pyrB*, *pyrC*, *pyrD*, *pyrE*) related to uracil synthesis, and Orotate phosphoribosyl transferase (OPRTase) encoded by *pyrE* is a key enzyme in pyrimidine biosynthesis (Ueda et al., 2009). *pyrE* gene mutants are resistant to 5-FOA but are uracil auxotrophs, and the selection system utilizing these properties has been successfully established (Ueda et al., 2009; Akasaka et al., 2013).

In our previous work, we investigated the effect of Bt on the gut microbiota diversity of the *Spodoptera exigua* (Li et al., 2022). However, the regulatory genes of Bt that interact with insect gut microbiota and the molecular mechanism of host immune regulation were not clear. In this study, we obtained an uracil auxotrophic strain, Bt GS57△*pyrE,* and clarified the role of uracil in activating the DUOX-ROS immune system and maintaining the stability of intestinal microbes. Our findings provide a theoretical basis for studying new insecticidal mechanisms of Bt.

## Materials and methods

### Insect rearing

*Spodoptera exigua* larvae were reared on an artificial diet following the method described previously, the main components are wheat germ powder, soybeans, and so on (Ren et al., 2013), and maintained at 26 ± 1°C with a relative humidity of 70 ± 10%.

### Bacterial strains, plasmids and growth conditions

The highly virulent strain of Bt GS57, known to be effective against *S. exigua* larvae, was maintained in our laboratory (Li et al., 2022). Competent Bt GS57 cells were prepared in 3.7% brain heart infusion medium, as per literature (Xu et al., 2020). Non-methylated plasmid DNA for transformation into *B. thuringiensis* cells was generated using *E. coli*. All *E. coli* strains were grown in LB medium. The pMAD and pSTK plasmids used in this study were obtained from Institute of Plant Protection, Chinese Academy of Agricultural Sciences.

### Bt GS57△*pyrE* strain construction

*B. thuringiensis* is an important biocontrol agent against various insect pests, including *S. exigua*. The *pyrE* gene plays a crucial role in regulating uracil synthesis in bacteria (Akasaka et al., 2013). To investigate the role of uracil synthesis in Bt virulence, a Bt GS57△*pyrE* mutant strain was constructed. In this study, the location of *pyrE* gene in the Bt GS57 genome was determined (Chromosome1:263694:264326), the 997-bp fragment upstream of *pyrE* (*pyrE* fragment A, *pyrE*A) and the 993-bp fragment downstream of *pyrE* (*pyrE* fragment B, *pyrE*B) were amplified from Bt GS57 genomic DNA with *pyrE*-1F/EU-RX and *pyrE*-1R/EU-FX primers, respectively. A 1.4 kb fragment of kanamycin resistance gene was amplified using EPK-F/R as primers and pSTK plasmid as templates. *pyrE*A, *kan*, and *pyrE*B were ligated together using overlapping PCR with primers *pyrE*-1F/R. The resulting fragment was inserted into the *Bam*H I-*Sal* I restriction sites of pMAD plasmids to construct the recombinant plasmid vector pMAD△*pyrE*, which was identified using PCR and DNA sequencing with pMAD-F and pMAD-R primers. The pMAD△*pyrE* plasmid was electroporated into competent Bt GS57 cells, and transformants were selected on LB agar plates based on erythromycin and kanamycin resistance. The *pyrE* gene deletion in the Bt GS57 was accomplished using homologous recombination as described previously (Janes and Stibitz, 2006; Liu et al., 2021). The Bt GS57△*pyrE* mutant was selected on LB agar plates containing kanamycin resistance without erythromycin and verified using PCR with primers *pyrE*-JF/R.

### Verification of Bt GS57△*pyrE* mutant strains

Uracil auxotrophs strains are often identified based on their resistance to 5-fluoroorotic acid (5-FOA), an antimetabolite commonly used for selection. To determine the working concentration for Bt GS57, LB broth medium was supplemented with 5-FOA at final concentrations of 0.1 mg/mL, 1.0 mg/mL, and 1.5 mg/mL. Subsequently, a single colony of Bt GS57 was inoculated into each of the media and grown for 24 h. Growth curves of Bt GS57 were generated for each concentration of 5-FOA. To assess the effectiveness of the selected concentration, a single colony of Bt GS57△*pyrE* and Bt GS57 was inoculated onto LB plates supplemented with the optimal concentration of 5-FOA. Growth of each strain on the plates was compared to determine the concentration at which 5-FOA effectively selects uracil auxotrophic mutants.

### Growth characteristics and biological activity assay of Bt GS57△*pyrE*

The crystal phenotypic and growth characteristics of Bt GS57△*pyrE* were assessed according to a previously published method (Li et al., 2022). Both Bt GS57△*pyrE* and Bt GS5*7* were inoculated into LB medium and grown at 30°C with shaking (200 rpm) for 30 h. The OD_600_ values were measured every 2 h to monitor the growth of Bt GS57△*pyrE,* and the results were compared with those of the Bt GS57 strain. The spore crystal mixtures of Bt GS57△*pyrE* and Bt GS57 were harvested and stained with 0.5% safranin, then observed using a light microscope (Olympus, U-HGLGPS, 100X oil objective) to evaluate their biological activity.

### Virulence assays

The Bt GS57△*pyrE* strain was cultured in 1/2 LB medium at 30°C for 46 h to obtain the spore crystal mixture. The mixture was then suspended in distilled water and diluted to gradient concentrations (0.05, 0.1, 0.2, 0.4, 0.8, 1.6 mg·ml^−1^). Subsequently, 100 μl of the diluted mixture was overlaid on a 33 mm diameter plastic tube containing artificial diet. The newly hatched larvae and early stage of 4th-instar larvae of *S. exigua,* which had been starved for 2 h, were then exposed to *B. thuringiensis* or 20 mM uracil (Sigma-Aldrich, St. Louis, United States) and reared under standard culture conditions, as described in a previous study (Li et al., 2022). The bioassays were repeated three times for each treatment, and mortality was recorded every 24 h. The semi-lethal concentration (LC_50_) was calculated after 72 h using Probit analysis (SPSS, Chicago, IL, United States).

### Quantification of gut bacteria by qPCR

Fourth instar larvae of *S. exigua* were fed with Bt GS57 or Bt GS57△*pyrE* strains at the semi-lethal concentration (3 mg cell crystal mixture per mL). The control group was added the sterile water. Genomic DNA from the gut, including contents, was extracted at various time points (0 h, 4 h, 8 h, 12 h and 24 h) using TIANamp Genomic DNA Kit (TIANGEN) following the manufacturer’s instructions. Quantitative PCR (qPCR) was performed using universal bacteria primers (16SF/R) (Table 1) to amplify 16S rRNA fragments with TB Green II Master Mix (TaKaRa Bio Inc. Japan). The expression levels of 16S rRNA were normalized using the *actin* gene as an endogenous control. All reactions were performed in triplicate, and the data were analyzed using the threshold cycle 2^−ΔΔCt^ method (Livak and Schmittgen, 2001).

**Table 1:** Primers used in this study.

Primer	(5′—3′) Primer sequence	Usage
EPK-F	AGTGGGAAGGTGTAACAGTATGACAGCGAACCATTTGAG	PCR of kanamycin resistance gene
EPK-R	GCGGTCGTCTTTTTTCGTGAAATTCCTCGTAGGC	
EU-RX	CTCAAATGGTTCGCTGTCATACTGTTACACCTTCCCACT	PCR of *pyrE* fragment
EU-FX	GCCTACGAGGAATTTCACGAAAAAAGACGACCGC	
*pyrE*-1F	CGCGGATCCTCCTGCGATTAAGCCAGTAG	overlapping PCR
*pyrE*-1R	ACGCGTCGACTATTCCTATCGCTGCACTCTTTTAT	
338F	ACTCCTACGGGAGGCAGCA	PCR of eubacterial 16S rRNA gene
806R	GGACTACHVGGGTWTCTAAT	
*Duox*-F	CGTATACTAATGGACCAGGTTTTCG	qPCR
*Duox*-R	AACCTGAGTCTTTGTGTACCTCC	
*PGRP*-F	CACTCGAACTTGCAATACGG	
*PGRP*-R	ACCAATCACGAACGAGGACC	
*Definsin*-F	GACTTGGACGCGAAATTGA	
*Definsin*-R	TGGTAGACAATGCTCCTGG	
*ceropin*-F	ACCAGCCTTGATGATACCG	
*ceropin*-R	ATGAAGTTCTCCCGAGTGTT	
*attacin*-F	CTGCCGAAAGTAAGACCAA	
*attacin*-R	AAGAACAAGGTGGGAGCAT	
16SF	TCCTACGGGAGGCAGCAGT	
16SR	GGACTACCAGGGTATCTAATCCTGTT	
*SeActin*-F	CTACCTCACGCCATTCTC	
*SeActin*-R	AACCTGAGTCTTTGTGTACCTCC	

### Bacterial composition and diversity of gut microbiota in *S. exigua* infected with Bt

Fourth instar *S. exigua* larvae were infected with Bt GS57 and Bt GS57△*pyrE* by spreading cell crystal mixture on the diet surface. The midguts of the infected larvae were dissected at five different time points (0 h, 4 h, 8 h,12 h and 24 h) and three replicates were prepared for each treatment, with 15 larvae per replicate mixed to obtain the samples. The DNA was extracted from the samples and amplified using the 338F/806R primer (Table 1) targeting the V3 and V4 hypervariable region of the bacterial 16S rDNA gene. The Illumina MiSeq platform was used for 16S rDNA gene amplicon sequencing by Beijing Biomarker Technologies company. The high-quality CCS (Circular Consensus Sequencing) sequence were obtained by removing the chimera (UCHIME, version 8.1) and clustering the resulting datasets as operational taxonomic units (OTUs) based on 97% similarity using USEARCH (version 10.0). The alpha diversity indices (ACE and Shannon) and beta-diversity (NMDS) were calculated for each sample, and differences in the microbial community structure between groups were analyzed using BMKCloud.^1^

### Analysis of *DUOX* and AMPs gene expression

The midguts of fourth instar larvae of *S. exigua* were collected at 0 h (control), 4 h, 8 h, 12 h and 24 h after Bt GS57 or Bt GS57△*pyrE* (3 mg/mL) infection, and total RNA was isolated from each sample. First-strand cDNA was synthesized from total RNA using the PrimeScript RT Reagent Kit with gDNA Eraser (Takara, China) following the manufacturer’s instructions. qPCR analysis was performed to analyze the influence of bacterial-derived uracil on immune-related genes. The housekeeping *actin* gene was used as endogenous control, and each reaction was performed in triplicate. The primers used are shown in Table 1. The results were calculated using the 2^−△△Ct^ method.

### Measurement of total ROS activity *in vivo*

The intestinal tissues of fourth instar larvae of *S. exigua* were dissected in pre-cooled PBS, ground in 500 μl PBS solution and centrifuged at 4°C and 3,000 g for 5 min. The supernatant was used for ROS levels detection following the instructions of the reactive oxygen species detection kit (Solarbio, China). Samples were incubated with 2′,7′-dichlorodihydrofluorescein diacetate (DCFH-DA) (10 μmol/L) for 30 min at 37°C in the dark and detected using a Tecan Infinite® M200 multiscan spectrum (Swiss) at excitation wavelengths of 488 nm and 525 nm to excite DCF. The fluorescence intensity of the final sample was calibrated with a negative control and three biological replicates were set for each treatment, with 15 larvae in each replicate.

### Data analysis

The results of gut bacterial load of *S. exigua* and the gene expression were tested for significance using Tukey’s HSD one-way ANOVA with SPSS (Statistical Package for the Social Sciences) Statistics 18.0 software. The different letters indicate that the difference is significant among groups at *p* < 0.05 level (*n =* 3).

## Results

### Identification and cloning of *pyrE* in Bt GS57

**Figure 1:**
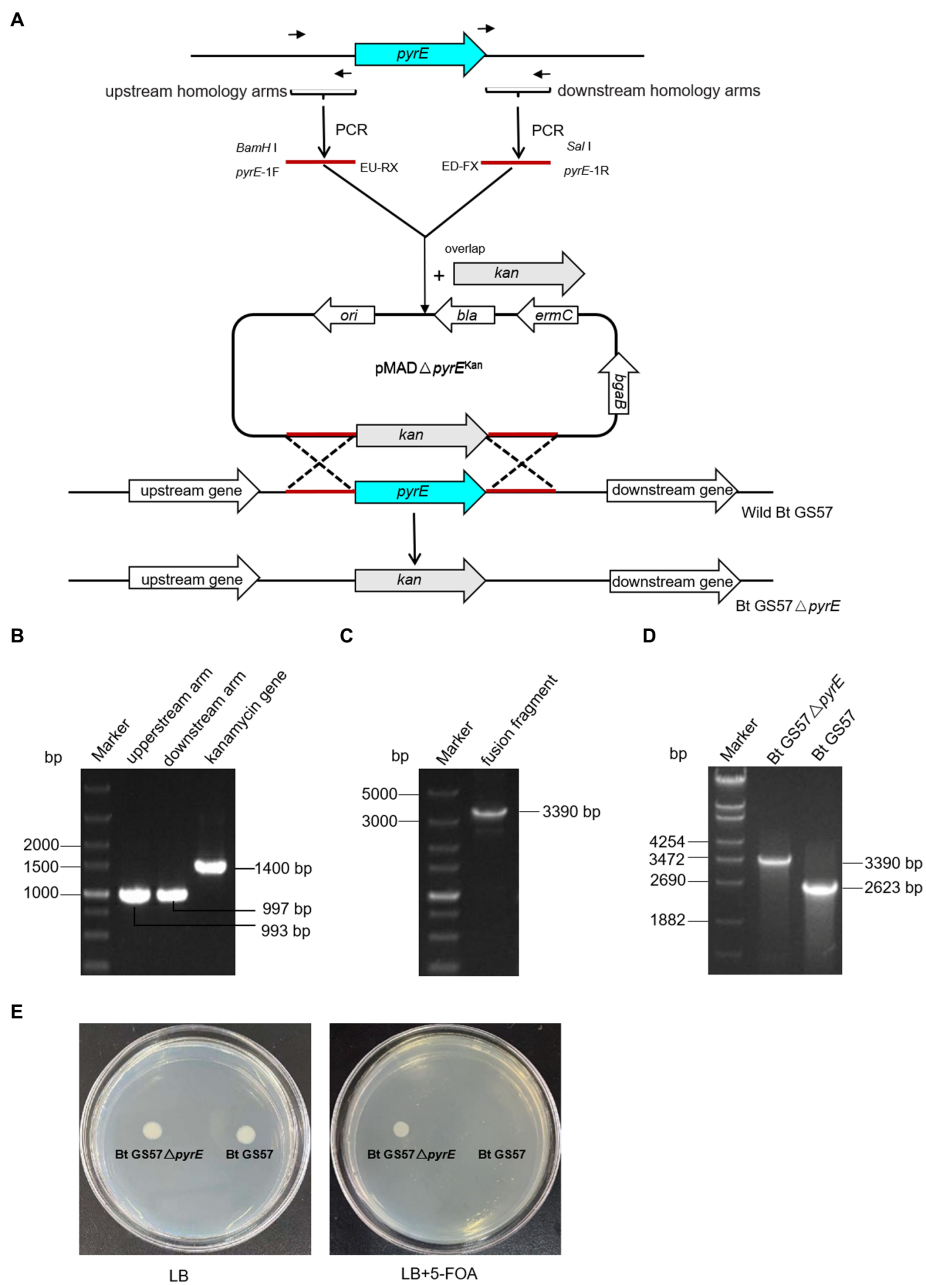
Construction and PCR identification of Bt GS57△*pyrE* mutant. **(A)** Bt GS57△*pyrE* constructed by homologous recombination, the *pyrE* was exchanged by kanamycin gene as a mutant. **(B)** PCR amplification of *pyrE* gene and kanamycin resistance gene. Marker: DL5000 Marker; the upstream homology arms fragment of *pyrE* gene amplified by *pyrE*-1F/EU-RX primer; the downstream homology arms fragment of *pyrE* gene amplified by ED-FX/*pyrE*-1R primer; the fragment of kanamycin resistance gene amplified by EPK-F/R primer. **(C)** The fusion fragment of upstream, downstream arms and kanamycin gene amplified by *pyrE*-1F/*pyrE*-2R primer. **(D)** PCR identification of Bt GS57△*pyrE* mutant. Marker: DL5000 Marker, the lane of Bt GS57△*pyrE* and Bt GS57 were *pyrE* fragment amplified by *pyrE*-JF/pyrE-JR prime. **(E)** Functional identification of the Bt GS57△ *pyrE* mutant, Bt GS57△*pyrE* grows normally on LB plates containing 5-FOA, while Bt GS57 does not.

The *pyrE* gene deletion mutant Bt GS57△*pyrE* was constructed by homologous recombination assay (Figure 1A), *pyrE* gene was identified in the genome of Bt GS57 (accession number NC_020238.1), located between 263,694 and 264,326 bp. A high amino acid identity of 99.52% was found between *pyrE* in *B. thuringiensis* strain GS57 and *Bacillus* sp. strain SYJ. The upstream and downstream homology arms of the *pyrE* gene (997-bp & 993-bp) from Bt GS57 strain, and a kanamycin resistance gene fragment (1400-bp) were amplified by PCR, respectively (Figure 1B). The overlapping PCR of the three fragments yielded a fusion fragment of 3,390 bp (Figure 1C).

The purified PCR product was cloned into the pMAD vector and transformed into *E. coli* SCS110. The recombinant pMAD-*pyrE* plasmid was then extracted and transformed into wild-type Bt GS57 by electro transformation. After two cycles of homologous recombination, the kanamycin gene replaced the *pyrE* gene, and the *pyrE* deletion mutant strain was constructed and verified by PCR (3,390 bp) and DNA sequencing (Figure 1D). Bt GS57△*pyrE* showed partial resistance to 5-FOA, as it grew in LB medium containing 1.5 mg/mL 5-FOA, while Bt GS57 could not (Figure 1E).

### Effects of *pyrE* deletion on biological characteristics and activity

**Figure 2:**
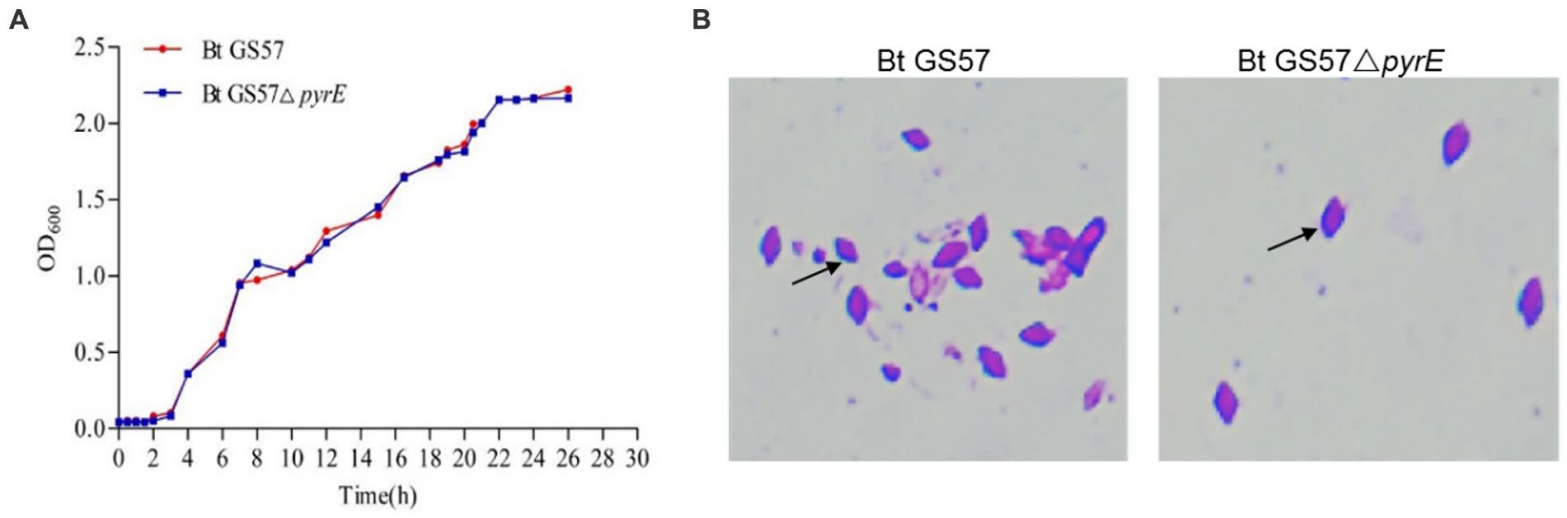
Growth curve and observation of crystal morphology of Bt GS57 and Bt GS57△*pyrE.* **(A)** Growth curve of Bt GS57 and Bt GS57△*pyrE*. **(B)** Observation of crystals by microscope, and the arrow points to the rhomboidal-shaped crystals.

**Figure 3:**
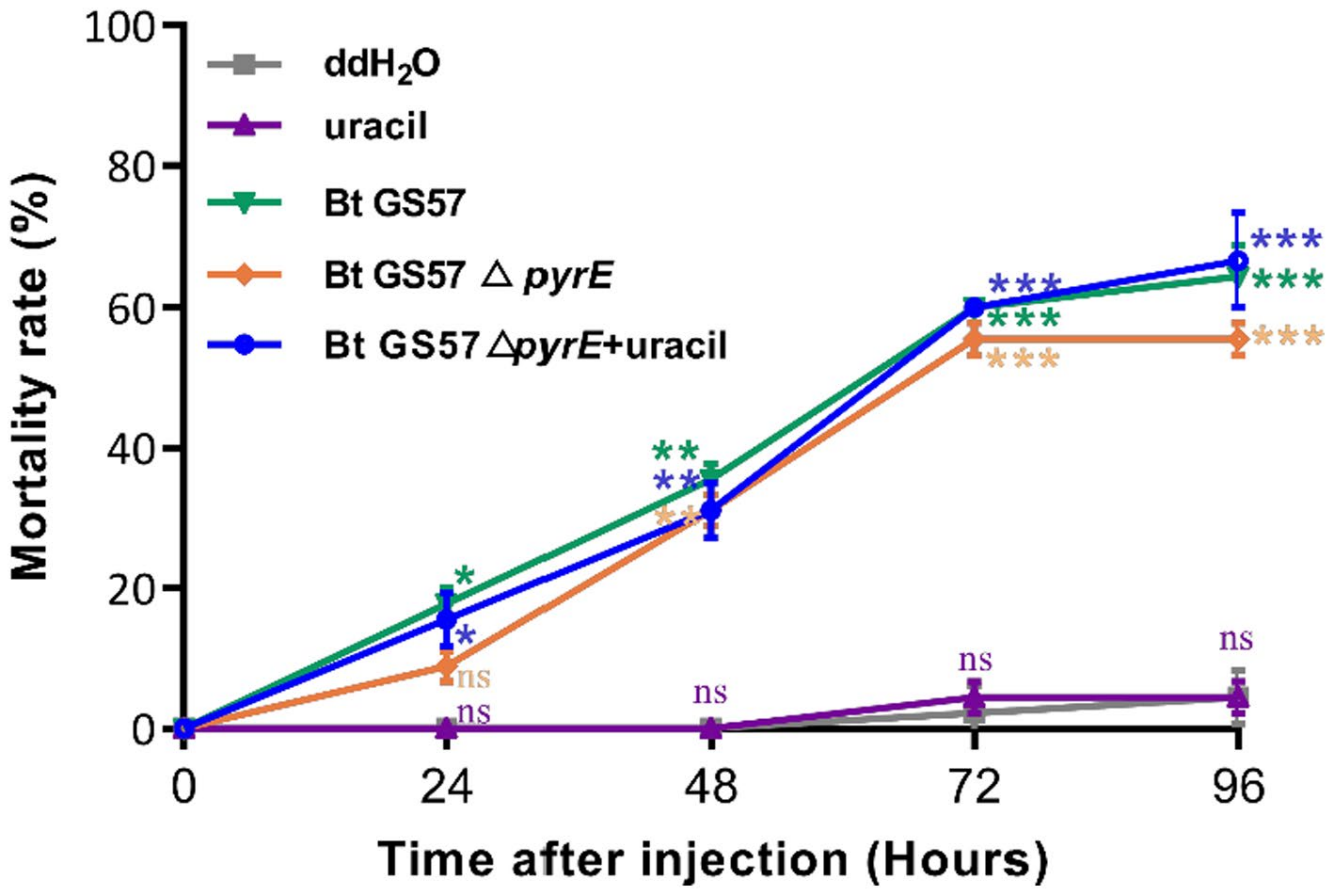
Mortality rate of *S. exigua* larvae feed with Bt strains at different times. The larvae were exposed to Bt GS57, Bt GS57△*pyrE* strains (3 mg·ml^−1^) and Bt GS57△*pyrE* + 20 mM uracil respectively, the control group was fed with ddH_2_O and 20 mM uracil (*n* = 15), repeat three times. The mortality rates of each group were analyzed every 24 h. Data are expressed as the mean ± SD from three independent experiments; asterisks indicate significant differences according to Student’s t-test, compared with the control value (*p < 0.05, **p < 0.01, ***p < 0.001).

To investigate the effects of *pyrE* deletion on biological characteristics and activity, Bt GS57 and Bt GS57△*pyrE* were cultured in 1/2 LB medium. The growth curves of Bt GS57 and Bt GS57△*pyrE* were similar, with a lag period of 0–3 h, a logarithmic growth period of 4–22 h, and a stable period (Figure 2A). Both strains produced spores and rhomboidal-shaped crystals at 44 h as assessed by microscope (Figure 2B). The bioassay results of Bt strains against the newly hatched *S. exigua* larvae showed that there was no significant change in LC_50_ of Bt GS57△*pyrE* (0.329 mg·ml^−1^) (Table 2). We also found that the mortality rate of the 4th-instar larvae of *S. exigua* after treat with Bt GS57△*pyrE* was approximately 56.67%, which is slightly lower than the mortality rate of 63.33% of Bt GS57 but is not significant. And the uracil supplement to Bt GS57△*pyrE* did not significantly changes the mortality rate (Figure 3). These results showed that *PyrE* deletion did not reduce the susceptibility of *S. exigua* larvae to Bt GS57.

**Table 2:** Bioassay of *Bacillus thuringiensis* isolates against the 1st instar larvae of *Spodoptera exigua.*

Bt isolate	Instar	Regression equation	LC_50_ (mg/ml)	95% Confidence limite
Bt GS57	1st instar larvae	y = 0.599 + 1.267x	0.337	0.214 ~ 0.501
Bt GS57△*pyrE*	1st instar larvae	y = 0.369 + 0.765x	0.329	0.169 ~ 0.563

LC_50_ is the concentration lethal to half of *S. exigua* larvae at 72 h. *N* = 15 (the number of larvae treated), biological repeat three times.

### Effects of Bt-derived uracil on the gut bacterial load of *S. exigua*

**Figure 4:**
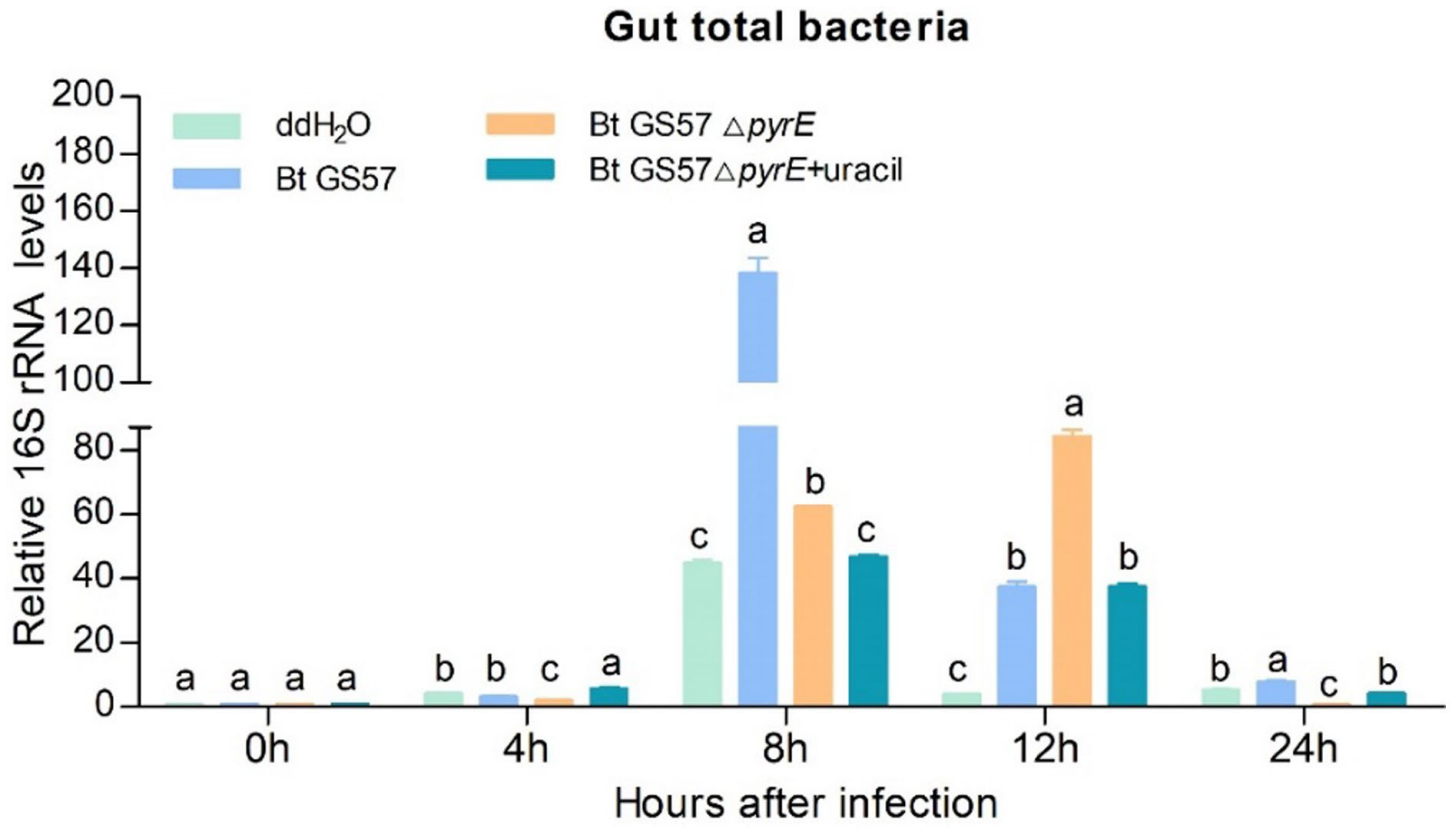
The effect of Bt GS57△*pyrE* on the bacterial load of the gut of *S. exigua.* mRNA levels of gut total bacteria in the 4th instar larvae of *S. exigua* (*n* = 3) following infection with Bt GS57, Bt GS57Δ*pyrE*, and Bt GS57Δ*pyrE* + uracil. Data are representative of three independent experiments (mean + s.e.m.). Significant differences between groups were determined by Tukey’s test as part of a one-way analysis of variance. Different lowercase letters (a, b or c) above bars indicate significant differences in the gut bacteria among groups (*p* < 0.05).

We investigate the effect of Bt GS57-derived uracil on the gut bacterial load of *S. exigua.* The gut bacterial loads of *S. exigua* were assessed at 4 h, 8 h, 12 h and 24 h post infection with Bt GS57△*pyrE* and Bt GS57, and compared to non-infected controls treated with ddH_2_O. qPCR analysis revealed that the gut bacterial load was upregulated in *S. exigua* after infection with both strains at different time intervals, compared to noninfected controls. However, at 8 h post-infection, the uracil deletion strain showed a significantly lower bacterial load than the wild-type strain. At 12 h, when the mutant was supplemented with uracil, there was no significant difference between the uracil deletion strain and the wild-type strain in terms of gut microbial load (Figure 4). These results suggest that Bt infection leads to dysbiosis of gut microbes and uracil is an important regulatory factor, we further investigation of the effect on immune genes.

### Bt GS57 infection causes dysbiosis of the gut microbiota

**Figure 5:**
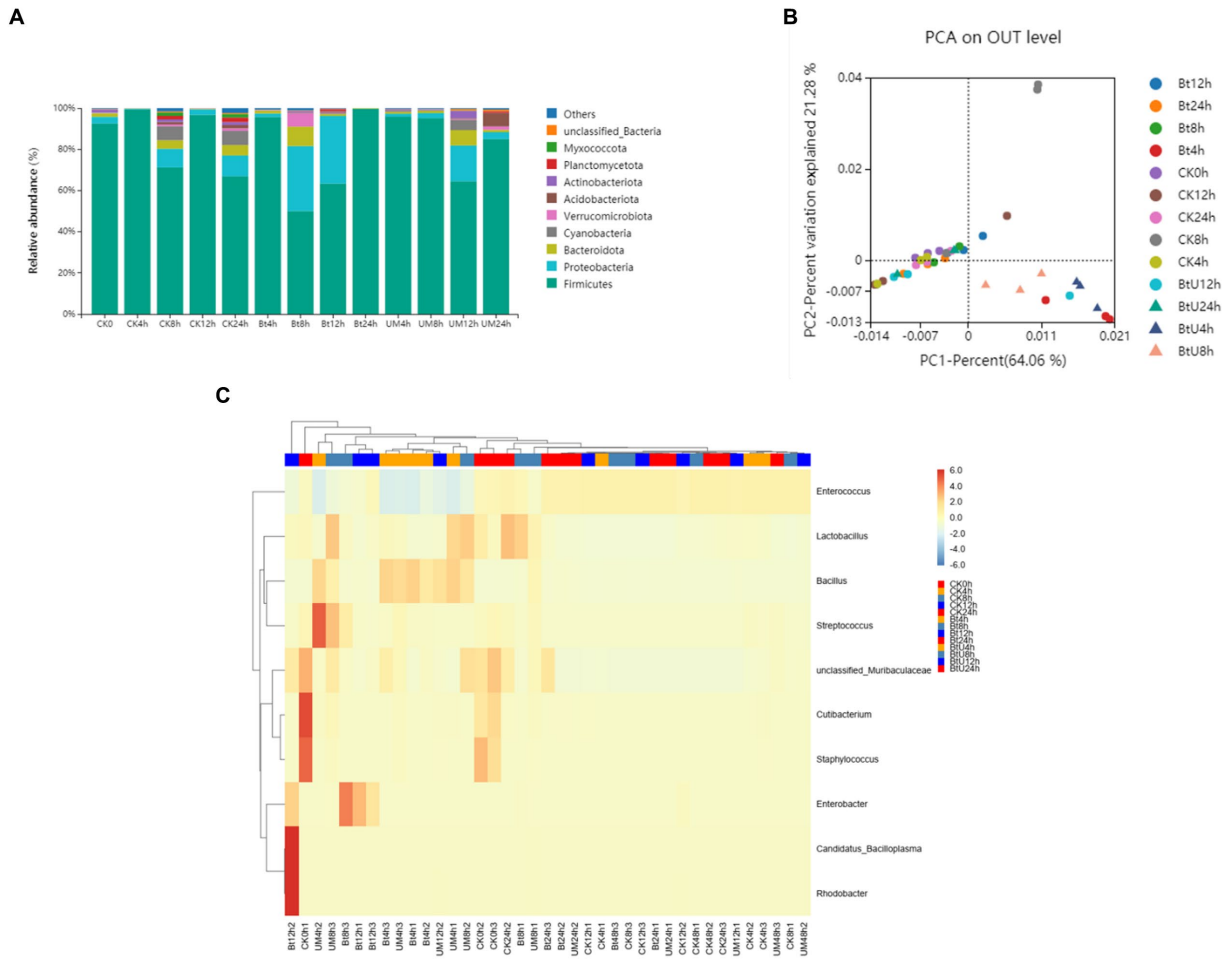
Gut bacterial dynamics in *S. exigua* after Bt GS57 and Bt GS57△*pyrE* infection. **(A)** Relative abundance of bacterial communities at the phylum level in different treatments. **(B)** PCA analysis of microbial communities according to host developmental stage (ANOSIM test, *p* = 0.001). **(C)** Heat map of major taxa at different treatments at the genus level generated by cluster analysis using the average method. Columns were clustered according to the similarity of bacterial abundance profiles. Each row represents an OUT assigned to the genus level. Color gradients represent the abundance variation of different species in the sample. Plotting scale, from red to blue, indicates the decrease in relative abundance of bacteria. Bt, Bt GS57△*pyrE*; UM, Bt GS57; CK, ddH_2_O.

To evaluate the impact of Bt GS57 infection on gut microbiota, we analyzed the midgut bacetria communities of Bt GS57-infected and Bt GS57**△***pyrE*-infected *S. exigua* larvae at different time intervals (0, 4, 8, 12, 24 h) using 16S rRNA gene sequencing (Table 1). We found that the abundance of Firmicutes and Proteobacteria changed dynamically at 8 h and 12 h post-infection5, and that the bacterial diversity decreased significantly in Bt GS57**△***pyrE* larvae compared to Bt GS57-infected larvae (Figure 5A), which Tsuggests that uracil produced by Bt GS57, may play a role in regulating gut microbiota composition. Principal Components Analysis (PCA) further revealed the deletion of uracil resulting in changes of gut microbial communities, which explained 21.28% of the variance in the group difference (Figure 5B). At the genus level, the relative abundances and distributions of bacterial families changed significantly in *S. exigua* after infection by Bt GS57 and Bt GS57△*pyrE* (Figure 5C), especially the genus Bacillus and Enterobacter (Supplementary Table S1). The results suggest that bacterial-derived uracil can alter gut bacterial composition and result in dysbiosis of the gut microbiota.

### Effects of the *pyrE* deletion on immune gene expression in the midgut

**Figure 6:**
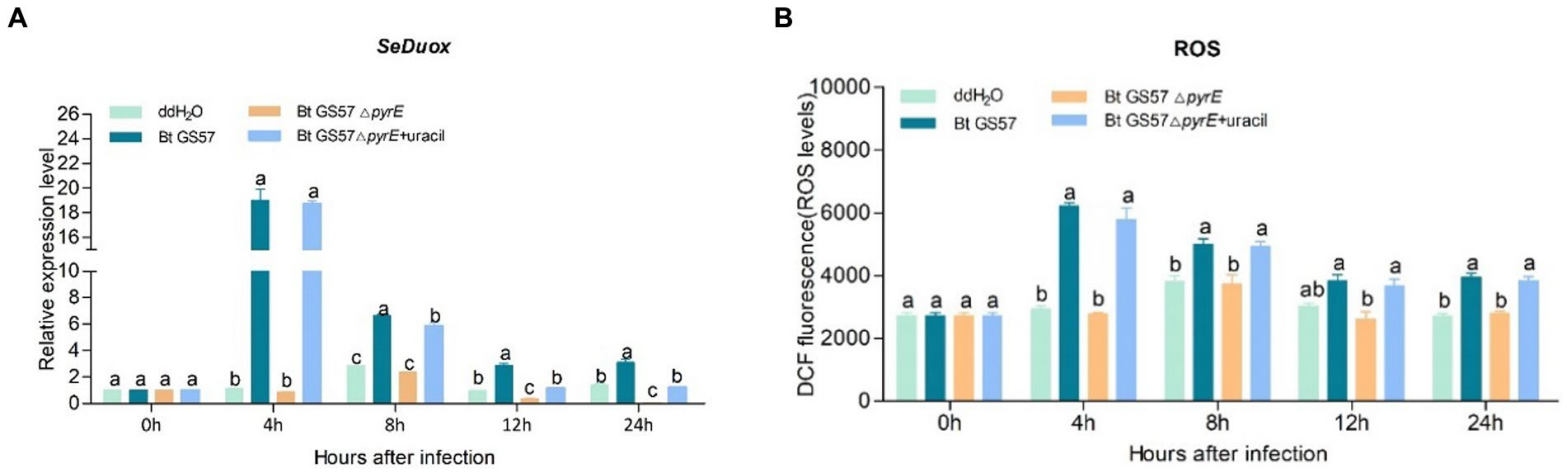
Effects of Bt GS57△*pyrE* on the expression of *DUOX* and ROS levels of *S. exigua*. **(A)** The expression of *SeDuox* gene of 4th instar larvae of *S. exigua* infection with Bt GS57, Bt GS57Δ*pyrE*, and Bt GS57Δ*pyrE* + uracil (*n* = 3). **(B)** Effects of Bt GS57△*pyrE* on The ROS level of 4th instar larvae of *S. exigua* infection with Bt GS57, Bt GS57Δ*pyrE*, and Bt GS57Δ*pyrE* + uracil (*n* = 3). Data are representative of three independent experiments (mean + s.e.m.). Different letters (a, b or c) indicate significant differences in the *DUOX* gene expression and ROS levels among different group as determined by Tukey’s test as part of a one-way analysis of variance (*p* < 0.05).

To test whether *pyrE* deletion could affect uracil deficient Bt strain (Bt GS57△pyrE) midgut immune responses, qPCR analysis was used to examine the effect of uracil on the expression of the *DUOX*, *PGRP-SA*, *attacin, defensin* and *ceropin* genes. *DUOX* gene expression and ROS levels was significantly upregulated after infection with Bt GS57, while Bt GS57△*pyrE* infection resulted in no significant difference compared to the ddH_2_O control at earlily time (4 h and 8 h), but a significant decrease compared to the wild strain, (Figure 6). Addition of uracil to Bt GS57△*pyrE* infected larvae restored *DUOX* and ROS expression to similar levels observed with Bt GS57 infection, indicating that uracil can activate the DUOX-ROS system and promote ROS production.

**Figure 7:**
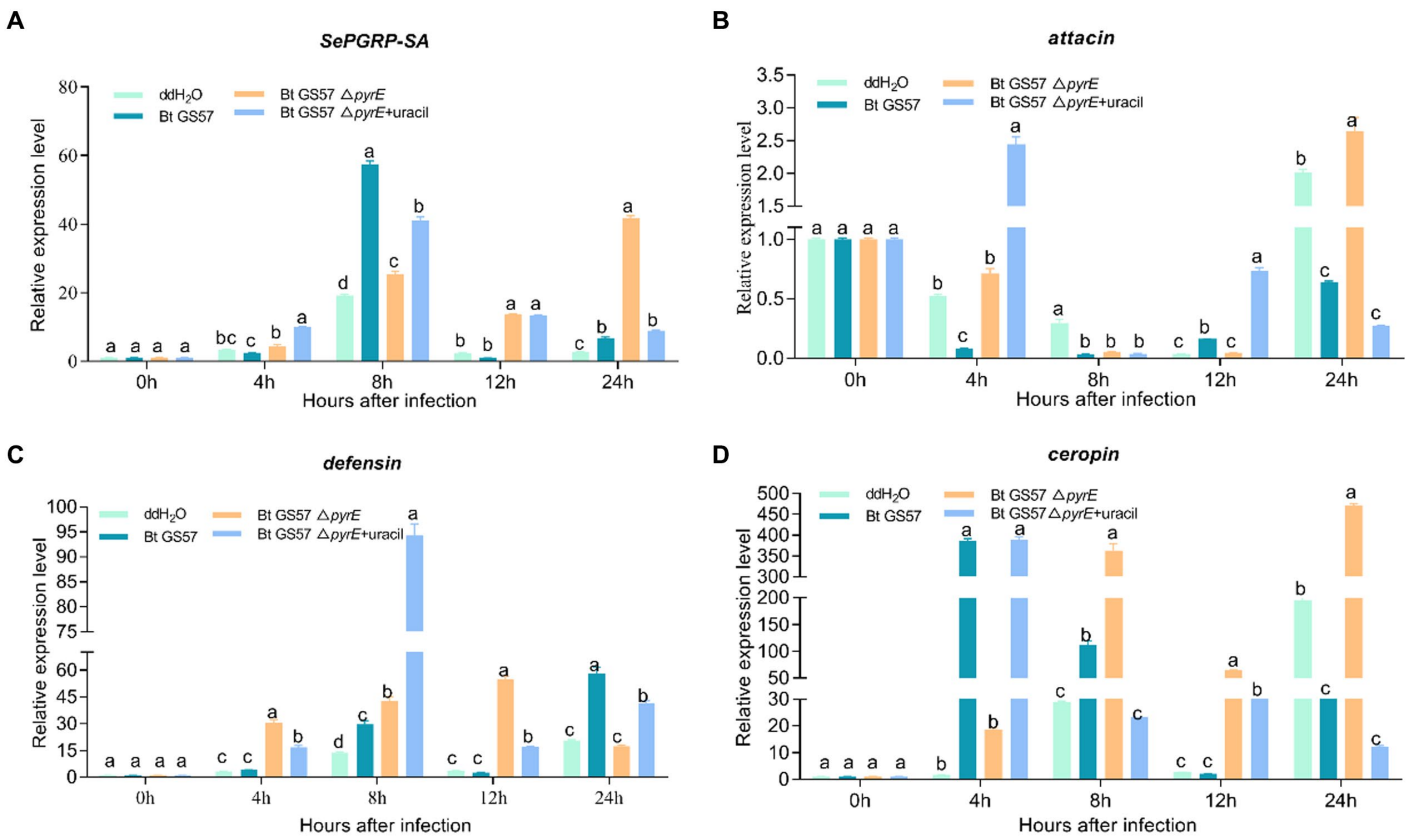
The effect of Bt GS57△*pyrE* on AMPs genes in the gut of *S. exigua*. **(A–D)** mRNA levels of *PGRP-SA*, *attacin, defensin* and *ceropin* in the 4th instar larvae of *S. exigua* (*n* = 3) following infection with Bt GS57, Bt GS57Δ*pyrE*, and Bt GS57Δ*pyrE* + uracil. Data are representative of three independent experiments (mean + s.e.m.). Significant differences between groups were determined by Tukey’s test as part of a one-way analysis of variance. Different letters (a, b or c) meant there was significant difference in the gene expression level among groups (*p* < 0.05).

Furthermore, qPCR analysis showed that the expression of *PGRP-SA* and AMP-related genes (*attacin, defensin* and *ceropin*) were significant different in the midgut of *S. exigua* infected by Bt GS57 and by Bt GS57△*pyrE* (Figure 7). *PGRP-SA, defensin* and *ceropin* was upregulated significantly in the midgut after infection with Bt GS57 and Bt GS57△*pyrE* at 8 h, while the *attacin* gene was downregulated which expressed at low levels.

## Discussion

The microbiota in the gut of insects play a vital role in the host’s development, immunity, and metabolism, as well as in the defence against pathogens (Engel and Moran, 2013; Siddiqui et al., 2022; Zhang et al., 2022). The balance between the gut microbial communities and the host’s immune system is critical for maintaining the health of the host. Insects rely on the ROS and AMPs pathway for their immune response, which regulates the intestinal flora and shows a synergistic effect in case of microbial disbalance (Kamareddine et al., 2018; Feng et al., 2020). Previous studies have shown that the joint action of metabolites of *B. thuringiensis* influences the midgut microbes (Engel and Moran, 2013). Additionally, recent research has demonstrated that uracil produced by pathogenic bacteria can upregulate the expression of the *DUOX* gene to produce ROS, which helps to resist pathogenic bacterial infection and maintain intestinal microbial homeostasis (Lee et al., 2015). In our previous research, we observed that gut microbiota can accelerate the killing of *S. exigua* by Bt GS57 (Li et al., 2022), which involves altered community structure and significantly decreased bacteria diversity. Building on this finding, we conducted further research to explore the effect of *B. thuringiensis* produced uracil on the interaction between intestinal microorganisms and immune genes. We constructed an uracil mutant by deleting the *pyre* gene in Bt GS57 and confirmed the characteristics of *pyrE* mutation. The results showed that the *pyrE* deletion mutant Bt GS57(**△***pyrE*) lost the ability to synthesize uracil and was resistant to 5-FOA (Figure 1). However, there were no significant changes in the biological function and growth characteristics of Bt GS57(**△***pyrE*) compared with the wild strain, and the crystal protein remained bipyramidal (Figure 2). Bioassay results also showed no significant effect on the toxicity of Bt GS57(Δ*pyrE*) against *S. exigua* larvae, indicating that the *pyrE* mutation did not change the strain’s characteristics (Table 2; Figure 3). Uracil, as a bacterial-derived ligand, plays a crucial role in activating the DUOX-dependent gut immune system, and uracil-modulated host signaling pathways are essential for DUOX-dependent ROS generation and host resistance to gut infection (Lee et al., 2015). In this study, we clarified the effect of uracil on activating the DUOX-ROS immune system and maintaining the stability of intestinal microbes. We found that after feeding on Bt GS57△*pyrE*, the *DUOX* expression and ROS levels were significantly down-regulated compared to the Bt GS57 (control) (Figure 6). However, after uracil supplementation, the expression of *DUOX* was significantly increased, inducing ROS generation, which suggests that the uracil secreted from Bt strain is possibly responsible for DUOX activation and could also induce intestinal ROS generation. These results show that uracil mediates the up-regulation of *DUOX* expression in the midgut, thereby increasing the production of midgut ROS levels.

In addition to ROS production, insects use the induction of AMPs as another defense mechanism to regulate their gut microbiota (Sheehan et al., 2018). The peptidoglycan recognition proteins (PGRPs) family acts as a pattern recognition receptor of the IMD or Toll signaling pathway and synthesizes AMPs, which play an essential role in the innate immunity of insects(Wang et al., 2019). Bt GS57△*pyrE* also induces an increase in PGRP and antimicrobial peptide-related genes (*defensin* and *ceropin*) at 8 h and 12 h compared with the sterile water control, but significantly lower than the wild strain (Figure 7), which showed the uracil also mediates the expression of PGRP and antimicrobial peptide-related genes. These results reveal that bacteria activate uracil-induced gut immune response, maybe act as a critical factor that determine gut microbe diversity and interactions with pathogenic bacteria. During the interaction between *B. thuringiensis* and gut microbes, the ROS and AMP response have a synergistic effect in resisting pathogenic bacterial infection and maintaining intestinal microbial homeostasis (Wei et al., 2017).

Most studies on the interaction between *B. thuringiensis* and insect midgut bacteria have focused on gut physiology, bacterial community structure, and composition. However, recent studies have shown that the toxicity of *B. thuringiensis* is associated with the gut microbiota. An investigation revealed that the diversity of bacterial microbiota in the tolerant *Aedes aegypti* larvae is reduced after Bt infection (Tetreau et al., 2018). In the Bt-resistant line of *Galleria mellonella*, Bt infection significantly reduced the diversity and abundance of the gut microbiota, possibly due to secreted antimicrobial factors in the resistant insects (Dubovskiy et al., 2016). The gut bacteria also affect Bt HD-1 insecticidal activity against *Plodia interpunctella* (Hübner) (Orozco-Flores et al., 2017). Similarly, Bt infection can cause a dynamic change in the gut microbiota of *S. exigua* (Li et al., 2022), and Bt Cry1Ac protoxin interacts with the gut microbiota to accelerate the mortality of *Plutella xylostella* larvae. However, the gut microbiota community composition of honeybees did not change significantly after feeding on transgenic Cry1Ah maize pollen (Jiang et al., 2013).

This study aimed to clarify the regulatory genes in the interaction between *B. thuringiensis* and insect resident midgut microflora. To investigate this, we compared the effect of *pyrE* deletion mutants of Bt GS57 on gut microbiota with the wild strain. It was found that the deletion of uracil could affect gut homeostasis, leading to an increase in bacteria and changes in bacterial community structure in the midgut of *S. exigua* larvae. The results suggest that the bacterial-derived uracil may trigger the DUOX-ROS immune response and influence the microbial communities of the gut, providing a better understanding of the interaction of *B. thuringiensis*-insect microbiota and exploring its insecticidal potential.

## Data availability statement

The datasets presented in this study can be found in online repositories. The names of the repository/repositories and accession number(s) can be found at: NCBI - PRJNA944960, SRR24031882-SRR24031841.

## Author contributions

DZ, HW, and WG conceived the research. DZ, HW, YL, QW, XG, and YJ conducted experiments. DZ and WG contributed material and secured funding. HW and YJ analysed data and conducted statistical analyses. DZ wrote the manuscript. All authors contributed to the article and approved the submitted version.

## Funding

This work was supported by the earmarked fund for Modern Agro-industry Technology Research System (CARS-13), the National Natural Science Foundation of China (Grant No. 31471775), the National Key Research and Development Program of China (2017YFD0200401), the Science and Technology Project of Hebei Education Department (QN2020119) and the Foundation of the Graduate School of the Chinese Academy of Agricultural Sciences (CAAS) (1610042022005).

## Conflict of interest

The authors declare that the research was conducted in the absence of any commercial or financial relationships that could be construed as a potential conflict of interest.

## Publisher’s note

All claims expressed in this article are solely those of the authors and do not necessarily represent those of their affiliated organizations, or those of the publisher, the editors and the reviewers. Any product that may be evaluated in this article, or claim that may be made by its manufacturer, is not guaranteed or endorsed by the publisher.

## Footnotes

^1^ www.biocloud.net

## References

[ref1] Akasaka N. Sakoda H. Hidese R. Ishii Y. Fujiwara S. (2013). An efficient method using *Gluconacetobacter europaeus* to reduce an unfavorable flavor compound, acetoin, in rice vinegar production. Appl. Environ. Microbiol. 79, 7334–7342. doi: 10.1128/AEM.02397-13, PMID: 24056455 PMC3837748

[ref2] Attila C. Ueda A. Wood T. K. (2009). 5-fluorouracil reduces biofilm formation in *Escherichia coli* K-12 through global regulator AriR as an antivirulence compound. Appl. Microbiol. Biotechnol. 82, 525–533. doi: 10.1007/s00253-009-1860-8, PMID: 19172264

[ref3] Bae Y. S. Choi M. K. Lee W. J. (2010). Dual oxidase in mucosal immunity and host-microbe homeostasis. Trends Immunol. 31, 278–287. doi: 10.1016/j.it.2010.05.003, PMID: 20579935

[ref4] Bai S. Yao Z. Raza M. F. Cai Z. Zhang H. (2021). Regulatory mechanisms of microbial homeostasis in insect gut. Insect Sci 28, 286–301. doi: 10.1111/1744-7917.12868, PMID: 32888254

[ref5] Broderick N. A. Raffa K. F. Handelsman J. (2006). Midgut bacteria required for *Bacillus thuringiensis* insecticidal activity. Proc. Natl. Acad. Sci. 103, 15196–15199. doi: 10.1073/pnas.0604865103, PMID: 17005725 PMC1622799

[ref6] Broderick N. A. Robinson C. J. McMahon M. D. Holt J. Handelsman J. Raffa K. F. (2009). Contributions of gut bacteria to *Bacillus thuringiensis*-induced mortality vary across a range of Lepidoptera. BMC Biol. 7:11. doi: 10.1186/1741-7007-7-11, PMID: 19261175 PMC2653032

[ref7] Dubovskiy I. M. Grizanova E. V. Whitten M. M. Mukherjee K. Greig C. Alikina T.. (2016). Immuno-physiological adaptations confer wax moth galleria mellonella resistance to *Bacillus thuringiensis*. Virulence 7, 860–870. doi: 10.1080/21505594.2016.1164367, PMID: 27029421 PMC5160394

[ref8] Engel P. Moran N. A. (2013). The gut microbiota of insects - diversity in structure and function. FEMS Microbiol. Rev. 37, 699–735. doi: 10.1111/1574-6976.12025, PMID: 23692388

[ref9] Feng M. Fei S. Xia J. Labropoulou V. Swevers L. Sun J. (2020). Antimicrobial peptides as potential antiviral factors in insect antiviral immune response. Front. Immunol. 11:2030. doi: 10.3389/fimmu.2020.02030, PMID: 32983149 PMC7492552

[ref10] Gao L. Song X. Wang J. (2020). Gut microbiota is essential in PGRP-LA regulated immune protection against plasmodium berghei infection. Parasit. Vectors 13:3. doi: 10.1186/s13071-019-3876-y, PMID: 31907025 PMC6945779

[ref11] Ha E. M. Lee K. A. Seo Y. Y. Kim S. H. Lim J. H. Oh B. H.. (2009). Coordination of multiple dual oxidase-regulatory pathways in responses to commensal and infectious microbes in drosophila gut. Nat. Immunol. 10, 949–957. doi: 10.1038/ni.1765, PMID: 19668222

[ref12] Ha E.-M. Oh C.-T. Bae Y. S. Lee W.-J. (2005). A direct role for dual oxidase in Drosophila gut immunity. Science 310, 847–850. doi: 10.1126/science.1117311, PMID: 16272120

[ref13] Janes B. K. Stibitz S. (2006). Routine markerless gene replacement in *Bacillus anthracis*. Infect. Immun. 74, 1949–1953. doi: 10.1128/IAI.74.3.1949-1953.2006, PMID: 16495572 PMC1418658

[ref14] Jiang W.-Y. Geng L.-L. Dai P.-L. Lang Z.-H. Shu C.-L. Lin Y.. (2013). The influence of Bt-transgenic maize pollen on the bacterial diversity in the Midgut of Chinese honeybees, *Apis cerana* cerana. J. Integr. Agric. 12, 474–482. doi: 10.1016/s2095-3119(13)60248-8

[ref15] Kamareddine L. Robins W. P. Berkey C. D. Mekalanos J. J. Watnick P. I. (2018). The Drosophila immune deficiency pathway modulates Enteroendocrine function and host metabolism. Cell Metab. 28, 449–462.e5. doi: 10.1016/j.cmet.2018.05.026, PMID: 29937377 PMC6125180

[ref16] Lee K. A. Kim B. Bhin J. Kim D. H. You H. Kim E. K.. (2015). Bacterial uracil modulates Drosophila DUOX-dependent gut immunity via hedgehog-induced signaling endosomes. Cell Host Microbe 17, 191–204. doi: 10.1016/j.chom.2014.12.012 25639794

[ref17] Lee K. A. Kim S. H. Kim E. K. Ha E. M. You H. Kim B.. (2013). Bacterial-derived uracil as a modulator of mucosal immunity and gut-microbe homeostasis in Drosophila. Cells 153, 797–811. doi: 10.1016/j.cell.2013.04.009, PMID: 23663779

[ref18] Li Y. Zhao D. Wu H. Ji Y. Liu Z. Guo X.. (2022). Bt GS57 interaction with gut microbiota accelerates *Spodoptera exigua* mortality. Front. Microbiol. 13:835227. doi: 10.3389/fmicb.2022.835227 35401496 PMC8989089

[ref19] Liu X. Zhang R. Hou S. Liu H. Wang J. Yu Q.. (2021). Identification and functional characterization of two homologous SpoVS proteins involved in sporulation of *Bacillus thuringiensis*.Pdf. Microbiol. Spectr 9:e0088121. doi: 10.1128/Spectrum.00881-21, PMID: 34612699 PMC8510167

[ref20] Livak K. J. Schmittgen T. D. (2001). Analysis of relative gene expression data using real-time quantitative PCR and the 2(-Delta Delta C(T)) method. Methods 25, 402–408. doi: 10.1006/meth.2001.1262 11846609

[ref21] Orozco-Flores A. A. Valadez-Lira J. A. Oppert B. Gomez-Flores R. Tamez-Guerra R. Rodriguez-Padilla C.. (2017). Regulation by gut bacteria of immune response, *Bacillus thuringiensis* susceptibility and hemolin expression in Plodia interpunctella. J. Insect Physiol. 98, 275–283. doi: 10.1016/j.jinsphys.2017.01.020 28167070

[ref22] Raymond B. Johnston P. R. Wright D. J. Ellis R. J. Crickmore N. Bonsall M. B. (2009). A mid-gut microbiota is not required for the pathogenicity of *Bacillus thuringiensis* to diamondback moth larvae. Environ. Microbiol. 11, 2556–2563. doi: 10.1111/j.1462-2920.2009.01980.x, PMID: 19555371

[ref23] Ren X. L. Chen R. R. Zhang Y. Ma Y. Cui J. J. Han Z. J.. (2013). A *Spodoptera exigua* cadherin serves as a putative receptor for *Bacillus thuringiensis* Cry1Ca toxin and shows differential enhancement of Cry1Ca and Cry1Ac toxicity. Appl. Environ. Microbiol. 79, 5576–5583. doi: 10.1128/AEM.01519-13, PMID: 23835184 PMC3754171

[ref24] Rinas U. H. K. Kang R. Seeger A. Schlieker H. (1995). Entry of *Escherichia coli* into stationary phase is indicated by endogenous and exogenous accumulation of nucleobases. Appl. Environ. Microbiol. 61, 4147–4151. doi: 10.1128/aem.61.12.4147-4151.1995, PMID: 8534082 PMC167726

[ref25] Ryu J. H. Ha E. M. Lee W. J. (2010). Innate immunity and gut-microbe mutualism in Drosophila. Dev. Comp. Immunol. 34, 369–376. doi: 10.1016/j.dci.2009.11.010, PMID: 19958789

[ref26] Sheehan G. Garvey A. Croke M. Kavanagh K. (2018). Innate humoral immune defences in mammals and insects: the same, with differences? Virulence 9, 1625–1639. doi: 10.1080/21505594.2018.1526531, PMID: 30257608 PMC7000196

[ref27] Shin S. C. Kim S. H. You H. Kim B. Kim A. C. Lee K. A.. (2011). Drosophila microbiome modulates host developmental and metabolic homeostasis via insulin signaling. Science 334, 670–674. doi: 10.1126/science.1212782, PMID: 22053049

[ref28] Siddiqui J. A. Khan M. M. Bamisile B. S. Hafeez M. Qasim M. Rasheed M. T.. (2022). Role of insect gut microbiota in pesticide degradation: a review. Front. Microbiol. 13:870462. doi: 10.3389/fmicb.2022.870462, PMID: 35591988 PMC9111541

[ref29] Tetreau G. Grizard S. Patil C. D. Tran F.-H. Tran Van V. Stalinski R.. (2018). Bacterial microbiota of *Aedes aegypti* mosquito larvae is altered by intoxication with *Bacillus thuringiensis* israelensis. Parasit. Vectors 11:121. doi: 10.1186/s13071-018-2741-8, PMID: 29499735 PMC5834902

[ref30] Ueda A. Attila C. Whiteley M. Wood T. K. (2009). Uracil influences quorum sensing and biofilm formation in Pseudomonas aeruginosa and fluorouracil is an antagonist. Microb. Biotechnol. 2, 62–74. doi: 10.1111/j.1751-7915.2008.00060.x, PMID: 21261882 PMC3815422

[ref31] Wang Q. Ren M. Liu X. Xia H. Chen K. (2019). Peptidoglycan recognition proteins in insect immunity. Mol. Immunol. 106, 69–76. doi: 10.1016/j.molimm.2018.12.021, PMID: 30590209

[ref32] Wei G. Lai Y. Wang G. Chen H. Li F. Wang S. (2017). Insect pathogenic fungus interacts with the gut microbiota to accelerate mosquito mortality. Proc. Natl. Acad. Sci. U. S. A. 114, 5994–5999. doi: 10.1073/pnas.1703546114, PMID: 28533370 PMC5468619

[ref33] Wu K. Yang B. Huang W. Dobens L. Song H. Ling E. (2016). Gut immunity in Lepidopteran insects. Dev. Comp. Immunol. 64, 65–74. doi: 10.1016/j.dci.2016.02.010 26872544

[ref34] Xu L. Han G. Fan X. Lv J. Zhang X. Peng Q.. (2020). Characteristics of the sigK deletion mutant from *Bacillus thuringiensis* var. israelensis strain Bt-59. Curr. Microbiol. 77, 3422–3429. doi: 10.1007/s00284-020-02150-9 32770390

[ref35] Zeng T. Jaffar S. Xu Y. Qi Y. (2022). The intestinal immune defense system in insects. Int. J. Mol. Sci. 23:15132. doi: 10.3390/ijms232315132, PMID: 36499457 PMC9740067

[ref36] Zhang X. Zhang F. Lu X. (2022). Diversity and functional roles of the gut microbiota in Lepidopteran insects. Microorganisms 10:1234. doi: 10.3390/microorganisms10061234, PMID: 35744751 PMC9231115

